# Femoral osteomyelitis caused by oral anaerobic bacteria with mixed bacteremia of *Campylobacter rectus* and *Parvimonas micra* in a chronic periodontitis patient: a case report

**DOI:** 10.1186/s12879-022-07573-2

**Published:** 2022-07-14

**Authors:** Naoya Itoh, Nana Akazawa, Yuichi Ishibana, Shunsuke Hamada, Sumitaka Hagiwara, Hiromi Murakami

**Affiliations:** 1 Division of Infectious Diseases, Aichi Cancer Center Hospital, 1-1 Kanokoden, Chikusa-ku, Nagoya, Aichi 464-8681 Japan; 2 Collaborative Chairs Emerging and Reemerging Infectious Diseases, National Center for Global Health and Medicine, Graduate School of Medicine, Tohoku University, 2-1 Seiryo-machi, Aoba-ku, Sendai, Miyagi 980-8575 Japan; 3 Department of Orthopedic Surgery, Aichi Cancer Center Hospital, 1-1 Kanokoden, Chikusa-ku, 464-8681 Nagoya, Aichi Japan; 4 Department of Head and Neck Surgery, Aichi Cancer Center Hospital, 1-1 Kanokoden, Chikusa-ku, Nagoya, Aichi 464-8681 Japan; 5 Aichi Cancer Center Hospital, 1-1 Kanokoden, Chikusa-ku, Nagoya, Aichi 464-8681 Japan

**Keywords:** Osteomyelitis, Chronic periodontitis, *Campylobacter rectus*, *Wolinella recta*, *Parbimonas micra*, *Fusobacterium nucleatum*

## Abstract

**Background:**

*Campylobacter* rectus is a gram-negative rod, and *Parvimonas micra* is a gram-positive coccus, both of which are oral anaerobes that cause chronic periodontitis. Chronic periodontitis can cause bacteremia and systemic diseases, including osteomyelitis. Hematogenous osteomyelitis caused by anaerobic bacteria is uncommon, and to date, there have been no reports of mixed bacteremia with *C. rectus* and *P. micra*. Here, we report the first case of osteomyelitis of the femur caused by anaerobic bacteria with mixed bacteremia of *C. rectus* and *P. micra* caused by chronic periodontitis.

**Case presentation:**

A 75-year-old man with chronic periodontitis, hyperuricemia, and benign prostatic hyperplasia was admitted to the hospital with a fracture of the left femur. The patient had left thigh pain for 4 weeks prior to admission. Left femoral intramedullary nail fixation was performed, and a large amount of abscess and necrotic tissue was found intraoperatively. The cultures of abscess specimens were identified as *P. micra*, *Fusobacterium nucleatum*, and *C. rectus*. *C. rectus* and *P. micra* were also isolated from blood cultures. *C. rectus* was identified by matrix-assisted laser desorption/ionization time-of-flight mass spectrometry and 16 S ribosomal RNA sequencing. Sulbactam-ampicillin was administered for approximately 1 month, after which it was replaced by oral clavulanic acid-amoxicillin for long-term suppressive treatment.

**Conclusions:**

Only five cases of bloodstream infection with *C. rectus* have been reported, and this is the first report of mixed bacteremia with *P. micra*. Clinicians should consider that chronic periodontitis caused by rare oral anaerobic bacteria can cause systemic infections, such as osteomyelitis.

## Background

Chronic periodontitis is a highly prevalent, dysbiosis-initiated, inflammatory condition that destroys the supporting tissues of the teeth [1]. While many types of microbiota are present in the oral cavity, only a limited number of species have been reported to be associated with chronic periodontitis [2, 3]. *Campylobacter rectus* (formerly known as *Wolinella recta*) is a gram-negative rod, and *Parvimonas micra* is a gram-positive coccus; both are oral anaerobes with virulence associated with periodontitis [1]. In common inflammatory diseases such as gingivitis and chronic periodontitis, the accumulation of plaque biofilm often triggers the proliferation and dilation of periodontal blood vessels, increasing their surface area and facilitating the entry of microorganisms into the bloodstream [4]. In most cases, the bacteremia is transient, but in some cases, the microorganisms disseminate to various target organs, causing subclinical, acute, or chronic infections. Although infectious endocarditis is a well-known complication of such odontogenic bacteremia, it can also cause central nervous system infections, respiratory infections, septicemia, and skeletal infections [4].

Hematogenous osteomyelitis caused by oral anaerobes is uncommon [5], and cases of *C. rectus* bacteremia are extremely rare; to date, there has been no report of a case of mixed bacteremia with *C. rectus* and *P. micra*. We report the first case of femoral osteomyelitis caused by oral anaerobic bacteria with mixed bacteremia of *C. rectus* and *P. micra* due to chronic periodontitis.

## Case presentation

A 75-year-old man with chronic periodontitis, hyperuricemia, and benign prostatic hyperplasia with a 4-week history of pain in his left thigh was admitted to the hospital. Six days prior to admission, he had fallen on the street and was admitted to a different hospital. He had a left femoral diaphyseal fracture, for which direct traction was performed. However, chest computed tomography (CT) showed a nodular shadow in the left upper lobe, and he was transferred to our cancer center because of possible bone metastasis of lung cancer. He had no smoking history or any other lung disease.

**Fig. 1:**
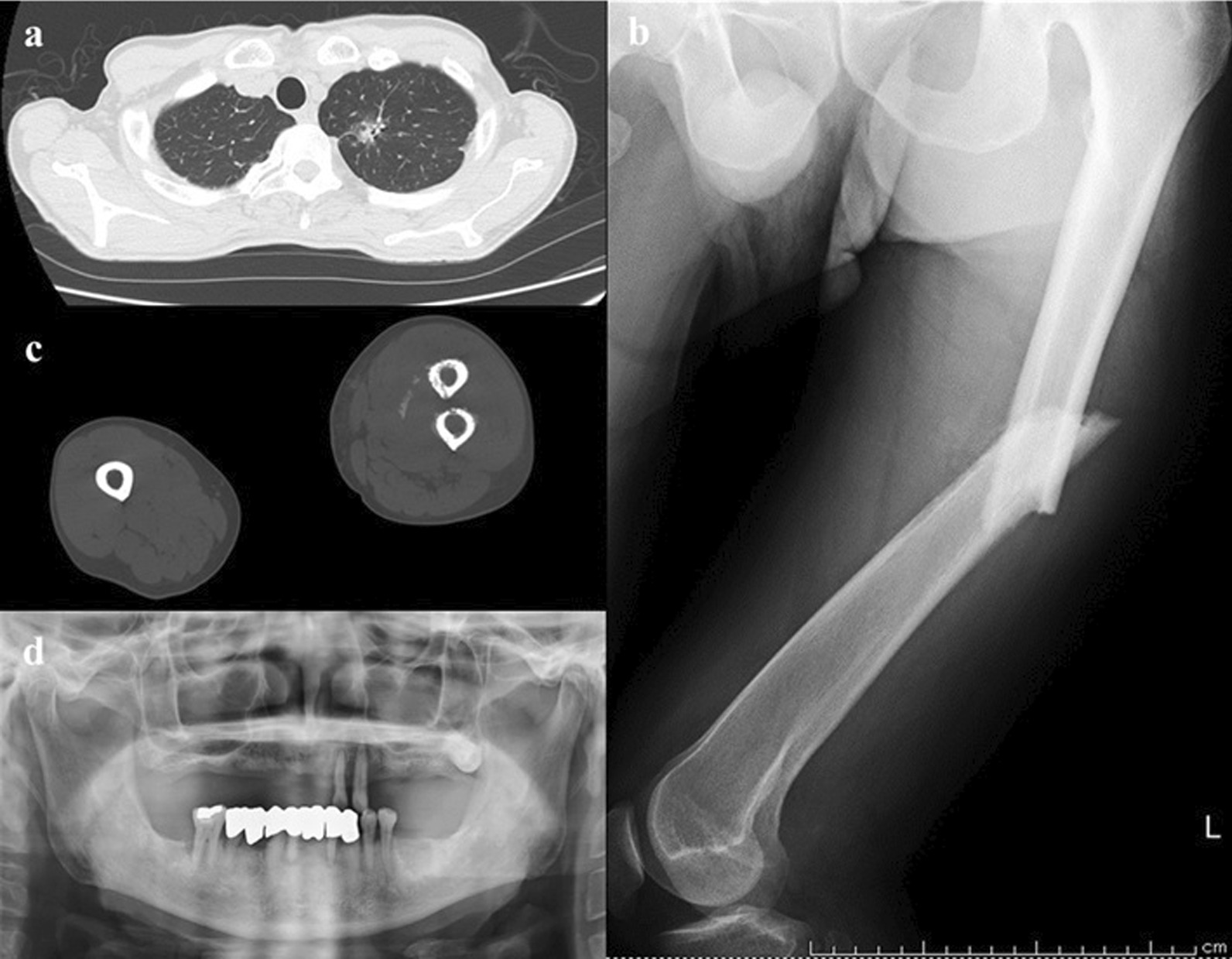
**a** Computed tomography scan of the thorax. A nodule with pleural indentation at the apex of the left lung was observed. **b** Preoperative X-ray. A fracture of the left femur with heterogeneous osteolytic changes of the bone cortex was noted. **c** Preoperative computed tomography scan of the lower extremity. A fractured left femur with heterogeneous osteolytic changes in the osteocortex was observed. **d** Panoramic images of the teeth. The third molar on the left maxilla had a slightly defined radiolucent area around the crown. Periradicular radiolucency of the left lateral incisor and canine of the maxilla and of the right first molar and left incisors of the mandible was observed

On examination, he appeared to be slightly ill. His body temperature was 36.8 °C, heart rate was regular at 91 bpm, blood pressure was 113/82 mmHg, respiratory rate was 20 bpm, and oxygen saturation was 96% in room air. Intraoral examination revealed poor oral hygiene and an impacted upper left third molar, indicating an 11 mm periodontal pocket, with plaque and bleeding. The patient’s left thigh was inflamed; however, his physical examination was otherwise unremarkable. Laboratory investigations revealed a white blood cell count of 13,040 cells/µL (normal: 3300–8600 cells/µL), neutrophil count of 12,062 cells/µL, eosinophil count of 652 cells/µL, hemoglobin level of 11.4 g/dL (normal: 13.7–16.8 g/dL), mean corpuscular volume of 89.9 fL (normal: 83.6–98.2 fL), hemoglobin A1c of 5.7% (normal: 4.9–6.0 g/dL), elevated serum levels of C-reactive protein at 26.7 mg/dL (normal: < 0.30 mg/dL), and alkaline phosphatase level of 308 U/L (normal 115–359 U/L). Human immunodeficiency virus (HIV)-1 and HIV-2 antibody tests came out negative. Analysis of the tumor markers showed 1.8 ng/mL carcinoembryonic antigen (normal: <5.0 ng/mL), 0.6 ng/mL squamous cell carcinoma (normal: < 1.5 ng/mL), 1.0 ng/mL cytokeratin fraction (normal: < 3.5 ng/mL), and 65.9 pg/mL progastrin-releasing peptide (normal: < 81.0 pg/mL). Chest radiography showed no abnormal findings, but chest CT showed a nodular shadow with pleural indentation in the left upper lobe (Fig. 1a). An X-ray (Fig. 1b) and CT (Fig. 1c) of the lower extremities showed a fracture of the left femur with heterogeneous osteolytic changes of the bone cortex. Panoramic images of the teeth showed a third molar on the left maxilla, with a slightly defined radiolucent area around the crown (Fig. 1d). Periradicular radiolucency of the left lateral incisor and canine of the maxilla and of the right first molar and left incisors of the mandible was observed.

**Fig. 2:**
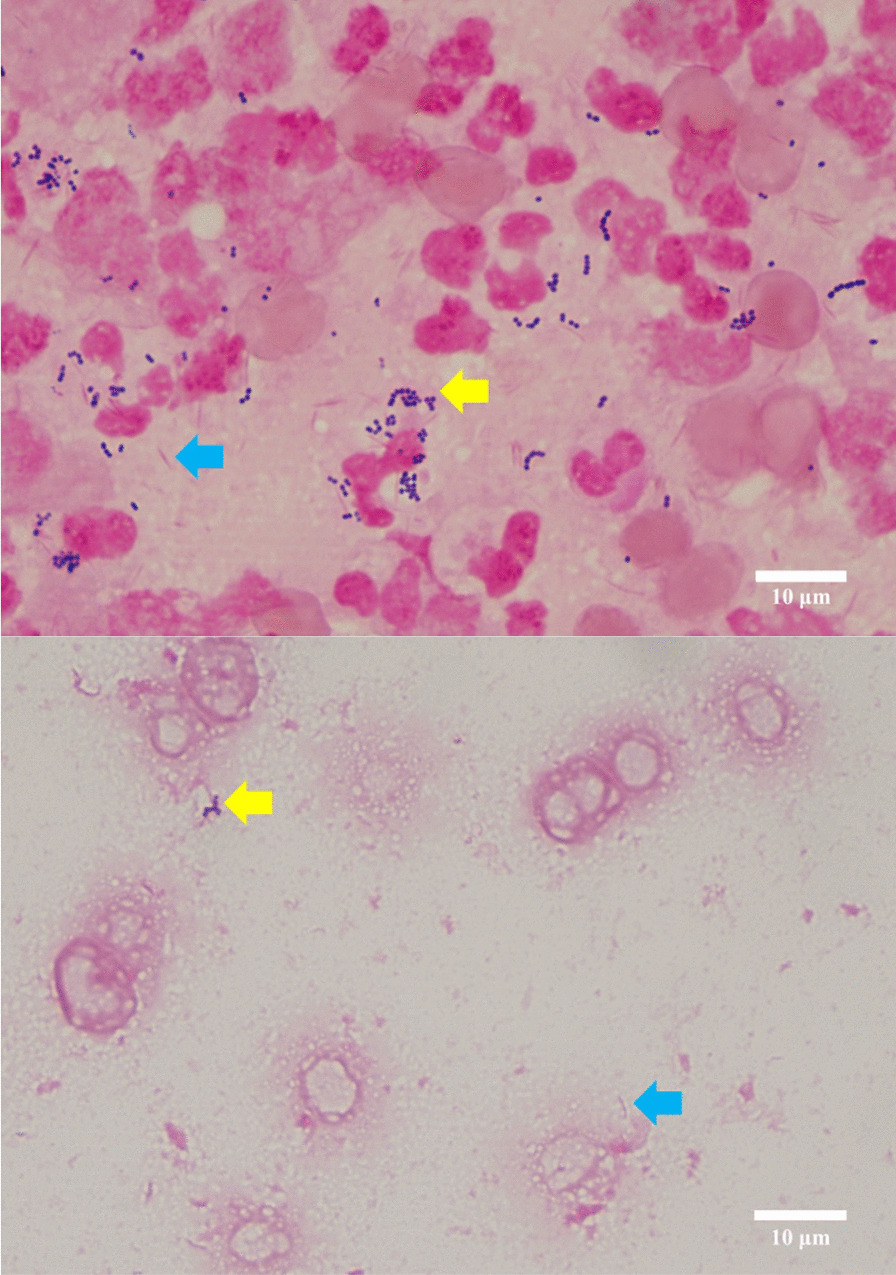
**a** Abscess shows numerous white blood cells, gram-negative (blue arrow) rods, and gram-positive cocci (yellow arrow). Gram staining, 1000 × (300 dpi). **b** Smear of blood culture demonstrating gram-negative rods (blue arrow) and gram-positive cocci (yellow arrow). Gram staining, 1000 × (300 dpi). (These images were acquired and captured using a Nikon eclipse 55i microscope (Nikon, Japan) and a Nikon Digital Color Camera Sight DS-Fi-1 (Nikon, Japan)

On the second day of admission (day 2), surgery was performed after a single dose of cefazolin (1 g) was administered as a perioperative prophylactic antimicrobial therapy. A substantial amount of abscess, hematoma, and necrotic tissue was detected within the vastus lateralis muscle and at the fracture site. Based on these findings, a pathological fracture due to osteomyelitis was strongly suspected. The left femur was debrided and irrigated, and left femoral intramedullary nail fixation was performed. The abscess revealed the presence of numerous white blood cells, gram-negative rods, and gram-positive cocci (Fig. 2a). A bone biopsy showed inflammation but no signs of malignancy.

**Fig. 3:**
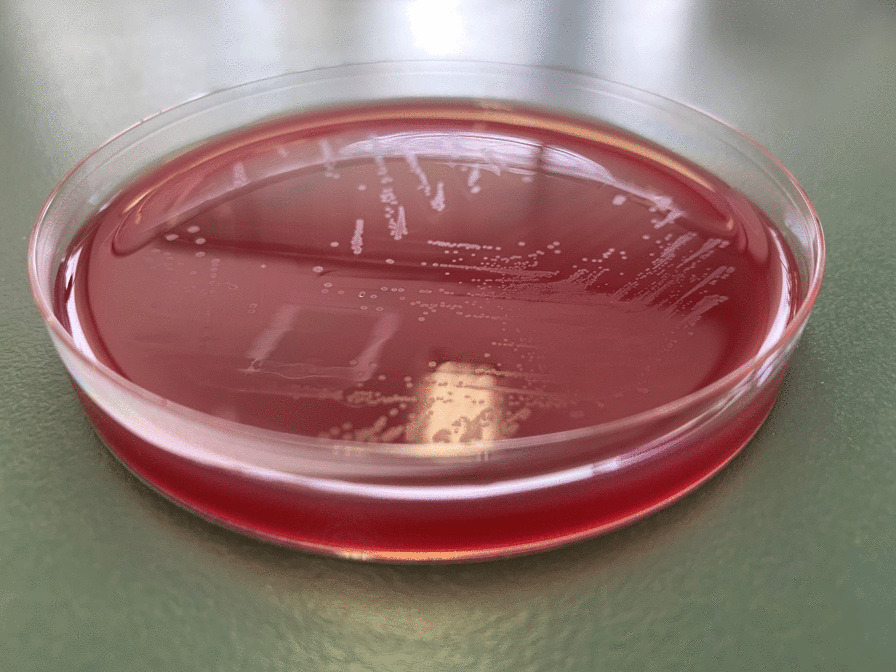
Translucent, rough, flat, non-hemolytic colonies after 48&nbsp;h on Brucella HK (RS) agar plate

Postoperatively, two sets of blood cultures were obtained in BACT/ALERT FA/FN PLUS culture bottles of the BacT/ALERT 3D system (Sysmex bioMérieux, Tokyo, Japan). After collecting the blood cultures, 3 g of sulbactam-ampicillin (SBT/ABPC) was administered every 6 h. The abscesses were cultured on a Sheep Blood Agar plate (Nissui Pharmaceuticals Co., Ltd., Tokyo), a chocolate agar EX II plate (Nissui Pharmaceutical Co., Ltd.), a MacConkey II agar plate (Becton Dickinson Co., Ltd., Tokyo), a Brucella HK (RS) agar plate (Kyokuto Pharmaceutical Co., Tokyo, Japan), a CHROMagar MRSA plate (Kanto Chemical Co, Inc., Tokyo, Japan), and a CHROMagar Candida plate (Becton Dickinson, Co, Inc., Tokyo, Japan). After 24 h of anaerobic incubation of the abscess specimens at 35 °C, small colonies of gram-positive cocci were observed on Brucella HK agar, and at 48 h, two types of small colonies of gram-negative rods were formed on the Brucella HK agar medium. The cultures in abscess specimens were identified as *Parvimonas micra* and *Fusobacterium nucleatum* using VITEK2 ver. 9.02 with VITEK 2 ANC ID card (SYSMEX bioMérieux Co., Ltd., Tokyo, Japan) with 97% and 99% probability, respectively. Because another form of gram-negative rod colony from the abscess specimen was not identified by VITEK2, it was evaluated using matrix-assisted laser desorption/ionization-time-of-flight mass spectrometry (MALDI-TOF MS) using the Vitek MS ver. 4.7.1 (bioMérieux, Tokyo, Japan), which demonstrated *Campylobacter rectus* with a 99.9% confidence value. The cultures of abscess specimen were negative for mycobacteria. For blood cultures at 95 h and 22 min of incubation, two FN bottles in the system showed a positive signal for bacterial growth, demonstrating gram-negative rods and gram-positive cocci (Fig. 2b). The positive bottles were subcultured on Sheep Blood Agar plate, MacConkey II agar plate, and Brucella HK (RS) agar plate. After 24 h of anaerobic incubation at 35 °C, small colonies of gram-positive cocci were observed on Brucella HK agar plate, and at 48 h, small gram-negative rods (Fig. 3) were formed on Brucella HK agar medium. Using the blood cultures, *P. micra* was identified by VITEK2 with 99% probability, and *C. rectus* was identified using Vitek MS ver. 4.7.1, with a 99.9% confidence value.

16 S ribosomal RNA (16 S rRNA) sequencing was performed on strains identified as *C. rectus* from abscesses. Based on a Basic Local Alignment Search Tool (BLAST) search of the sequence on the 16 S rRNA gene of the isolated strain, the homology with the standard strain *C. rectus canine* oral taxon 010 clone OB004 (GenBank Accession No.: JN713167.1) was 99.02% (1610/1626 bp). Blood cultures and abscesses of *C. rectus* and *F. nucleatum* were poorly developed, making it difficult to perform antimicrobial susceptibility testing. According to the methodology recommended by the Clinical and Laboratory Standards Institute (CLSI), document M100-S25 (2015) minimal inhibitory concentration (MIC) breakpoints for the *Bacteroides fragilis* group, the MIC of antimicrobial agents determined by broth microdilution for *P. micra* isolates from blood cultures and abscess specimens were as follows: penicillin G, ≤ 0.06 mg/L; ampicillin, ≤ 0.13 mg/L; clindamycin, 0.25 mg/L; cefmetazole, ≤ 16.0 mg/L; and meropenem, ≤ 0.25 mg/L. Sputum culture showed the presence of only alpha-hemolytic streptococci, *Neisseria* spp., and *Haemophilus* spp. Three series of sputum samples for mycobacterium were negative. Transthoracic echocardiography showed no obvious vegetation. The final diagnosis was pathological fracture and hematogenous osteomyelitis due to *P. micra*, *F. nucleatum*, and *C. rectus* caused by periodontitis.

Postoperatively, the patient was provided dental care and chronic periodontitis examination by a dentist. The pain in his left thigh gradually reduced, and he was able to walk with a cane. SBT/ABPC was administered for 27 days and subsequently changed to oral amoxicillin clavulanic acid (1500 mg/day) on day 29. The patient was discharged on day 33. Because of the residual intramedullary nail, antimicrobial agents were prescribed for long-term suppressive therapy. The left lung nodule was suspected to be a primary lung cancer, and we plan to investigate it at our hospital.

## Discussion and conclusions

We report a case of osteomyelitis caused by anaerobic bacteria with mixed bacteremia of *C. rectus* and *P. micra* as a result of chronic periodontitis. Osteomyelitis caused by anaerobic bacteria is rare in adults, and this is the first case of mixed bacteremia with *C. rectus* and *P. micra*. In addition, this case is educational for clinicians in that it provides insights into how poor oral hygiene can lead to systemic diseases.

Hematogenous osteomyelitis is more common in prepubertal children and primarily involves the metaphyseal ends of the long bones (especially the tibia and femur), most often as a single focus [5]. Vertebral bodies are the most common sites of hematogenous osteomyelitis in adults, but osteomyelitis of the long bones is rare. In adults, the most common species involved in hematogenous osteomyelitis is *Staphylococcus aureus* but occasionally includes *Enterobacter* spp. and streptococci [5, 6]. Anaerobic osteomyelitis is rare and usually arises from infections of the adjacent soft tissues [7]. The role of anaerobes is increasingly being recognized in the bacteriology of osteomyelitis, but the prevalence of anaerobes in this disease is unknown. Previous cases of osteomyelitis caused by anaerobic bacteria have been mainly reported in children and relatively rarely in adults [6, 8]. Most patients with osteomyelitis caused by anaerobic bacteria are infected with anaerobic bacteria elsewhere in the body, which is the source of the organisms involved in osteomyelitis [6]. In this case, transthoracic echocardiography was negative for infective endocarditis, suggesting that septic embolization from the heart valve was unlikely to have caused the osteomyelitis. Our patient had chronic periodontitis, and oral anaerobes were identified in abscesses and blood cultures, indicating hematogenous osteomyelitis of the femur originating from oral disease. Cases of distal embolization have often been reported in interventions related to atherosclerotic lesions of the femoral artery [9]. This patient had no history of smoking, hypertension, or dyslipidemia, which are risk factors for atherosclerosis, and did not undergo screening tests for atherosclerosis. However, the male, old, and hyperuricemic status was considered to have possibly contributed to atherosclerosis of the femoral artery, which might have served as a nidus for infection at the site [10, 11].

*C. rectus* is an anaerobic gram-negative rod, first described in 1981 as *Wolinella recta*; it was transferred to the genus *Campylobacter* in 1991 based on phylogenetic studies [12]. *P. micra* is a gram-positive anaerobic bacterium that was previously classified in the genus *Peptostreptococcus* but was reclassified as a member of the genus *Micromonas* in 1999 and as a member of the genus *Parvimonas* in 2006 [13]. *F. nucleatum* is an anaerobic gram-negative rod [14]. These microorganisms are members of the oral commensal flora. In our case, *P. micra* and *F. nucleatum* were identified by VITEK 2, but *C. rectus* was difficult to identify; thus, MALDI-TOF MS and 16 S rRNA sequencing were used. *C. rectus* is a difficult bacterium to identify and culture and is often identified by sequencing of the 16 S rRNA gene (Table 1) [15–19]. However, Genderini et al. identified *C. rectus* by MALDI-TOF MS and 16 S RNA sequencing and reported that MALDI-TOF MS is a rapid and reliable alternative to expensive molecular techniques such as 16 S rRNA sequencing [19]. In our case, the results of MALDI-TOF MS and 16 S RNA sequencing were consistent with *C. rectus*, and MALDI-TOF MS was considered a reliable method.

**Table 1:** Summary of published cases of blood stream infection due to *Campylobacter rectus*

Author, country, year, reference	Age (years), sex	Symptoms	Underlying condition	Diagnostic method	Susceptibility MIC (µg/mL)	Other bacteria isolated	Treatment	Outcome
Lam et al. Hong Kong, 2011, [15]	41, F	Headache, ptosis, diplopia	Subdural empyema, mycotic intracranial aneurysm	16 S rRNA sequencing	ND	none	Coil embolization, craniectomy, VCM, CTRX, until dead	Died
Leo and Bolger USA, 2014, [16]	55, M	Headache, ophthalmoplegia, ptosis, fever	Septic cavernous sinus thrombosis, dental caries	16 S rRNA sequencing	ND	none	Anticoagulation, steroids, VCM, CLDM, TAZ/PIPC (unknown duration)	Survived
Jawad et al., UK, 2018, [17]	29, M	Sore throat, fever, myalgia	Lemierre’s syndrome, septic pulmonary embolism, G6PD deficiency	MALDI-TOF MS, 16 S rRNA sequencing	ND	none	Anticoagulation, CVA/AMPC, CAM, TAZ/PIPC, MNZ, 6 weeks	Survived
Oka et al. Japan, 2018, [18]	70, F	Headache, fever	Clival osteomyelitis, cavernous sinus thrombosis, septic pulmonary embolism, odontogenic infection, HTN	unknown	ND	*Fusobacterium nucleatum*	SBT/ABPC, fluconazole, MEPM, MNZ (unknown duration)	Survived
Genderini et al. Belgium, 2019, [19]	70, M	Weight loss, fatigue, dysphagia, fever, cough, dyspnea	HSV-1 esophagitis, HIV-1 infection, periodontitis	MALDI-TOF MS, 16 S rRNA sequencing	CVA/AMPC 0.047	*Solobacterium moorei*	CVA/AMPC (unknown duration)	Survived
Present case	75, M	Thigh pain	Femoral osteomyelitis, periodontitis, hyperuricemia, benign prostatic hyperplasia	MALDI-TOF MS, 16 S rRNA sequencing	ND	*Parvimonas micra*	SBT/ABPC, CVA/AMPC	Survived

MIC, minimum inhibitory concentration; F, female; M, male; ND, not done; rRNA, ribosomal RNA; VCM, vancomycin; CTRX, ceftriaxone; CLDM, clindamycin; TAZ/PIPC, tazobactam/piperacillin; G6PD, glucose-6-phosphate dehydrogenase; MALDI-TOF MS, matrix-assisted laser desorption/ionization-time-of-flight mass spectrometry; CVA/AMPC, clavulanic acid/amoxicillin; CAM, clarithromycin; MNZ, metronidazole; HTN, hypertension; SBT/ABPC, sulbactam/ampicillin; MEPM, meropenem; HIV, human immunodeficiency virus; HSV, herpes simplex virus

*C. rectus* bacteremia cases are extremely rare, with only five cases reported as of date (Table 1) [15–19]. Furthermore, only two cases of mixed bacteremia have been reported, and ours is the first case of mixed bacteremia with *P. micra*. This finding may be attributed to the aforementioned difficulties with conventional diagnostic methods and to the advances in diagnostic technology.

Chronic osteomyelitis usually develops more than 6 weeks after bone infection and is characterized by bone destruction and sequestra formation [20]. Our patient was diagnosed with chronic osteomyelitis because he had experienced thigh pain for at least 4 weeks, and abscess formation was observed. Management of chronic osteomyelitis requires surgical and medical approaches, including aggressive surgical debridement and subsequent long-term antibiotic therapy [21]. There is a lack of clinical randomized trials evaluating the most appropriate agents, routes of administration, and duration of treatment for chronic osteomyelitis [22]. Many experts support continuing intravenous antibiotics for at least 4–6 weeks after surgical debridement, which is considered as the time it takes for the debrided bone to be covered by vascularized soft tissue. Long-term antibiotic therapy (3–6 months) with oral antimicrobials is usually required because of the poor transfer of antimicrobials into bone tissue and high recurrence rate. Patients who manifest inadequate surgical debridement or who cannot be treated surgically are generally treated with long-term antibiotic suppression therapy, similar to the treatment of joint prosthesis infections when removal of the prosthesis is not possible [23–26]. In our case, the susceptibility of *P. micra* to antimicrobial agents was revealed, but the susceptibility of *C. rectus* and *F. nucleatum* was unknown. In a recent epidemiological survey of susceptibility of *Fusobacterium* spp. in Japan (including *F. nucleatum*), all strains were 100% susceptible to ampicillin and SBT/ABPC [27, 28]. Data on the antibiotic susceptibility of *C. rectus* are scarce, and there are currently no accepted guidelines for testing or interpretation of results [15]. However, limited studies have shown that *C. rectus* is susceptible to CVA/AMPC. There are no reports of beta-lactamase production by *C. rectus* [29]. Our patient was treated with SBT/ABPC IV for 4 weeks and switched to oral CVA/AMPC for long-term suppressive therapy due to the presence of a prosthesis.

In conclusion, we report the first case of osteomyelitis caused by anaerobic bacteria with bacteremia of *C. rectus* and *P. micra* associated with chronic periodontitis. Clinicians should consider that chronic periodontitis can result in systemic infection caused by rare oral pathogens.

## Data Availability

The data used and/or analyzed during the current study are available from the corresponding author upon reasonable request.

## Abbreviations

SBT/ABPC: Sulbactam-ampicillin
MALDI-TOF MS: Matrix-assisted laser desorption/ionization time-of-flight mass spectrometry
MIC: Minimal inhibitory concentration
CVA/AMPC: Amoxicillin clavulanic acid
rRNA: Ribosomal RNA
